# Rutin (quercetin-3-rutinoside) modulates the hemostatic disturbances and redox imbalance induced by *Bothrops jararaca* snake venom in mice

**DOI:** 10.1371/journal.pntd.0006774

**Published:** 2018-10-11

**Authors:** Ana Teresa Azevedo Sachetto, Jaqueline Gomes Rosa, Marcelo Larami Santoro

**Affiliations:** 1 Laboratory of Pathophysiology, Institute Butantan, São Paulo, São Paulo, Brazil; 2 Department of Medical Sciences, School of Medicine, University of São Paulo, São Paulo, São Paulo, Brazil; Muséum National d'Histoire Naturelle, FRANCE

## Abstract

Snakebites are a major Collective Health problem worldwide. In Brazil, *Bothrops jararaca* snake venom (BjV) evokes hemostatic disturbances, bleeding manifestations, and redox status imbalance. Specific antivenom therapy, although efficacious to revert most snakebite-induced manifestations, is incapable of treating secondary manifestations, such as oxidative/nitrosative stress. Searching for new complementary therapies that could attenuate physiological derangements triggered by envenomation, we elected to test quercetin-3-rutinoside (rutin) by its potential as both a potent antioxidant and a hemostasis modulatory compound. The activity of rutin was evaluated both on the biological activities of crude BjV *in vitro*, and *in vivo* by the ability of rutin (14.4 mg/kg b.w.) to modulate hematological, hemostatic and redox status markers altered by BjV injection (1.6 mg/kg b.w., s.c.) in mice. *In vitro*, rutin failed to inhibit BjV-induced platelet aggregation and biological activities of major BjV enzymes (metalloproteinases, phospholipases A_2_, serine proteases, and L-amino acid oxidases). On the other hand, rutin attenuated local hemorrhage, and the increase in reactive species, prevented the fall in RBC counts and fibrinogen levels, diminished tail bleeding and shortened prothrombin time (PT) evoked by envenomation. Furthermore, rutin reduced tissue factor (TF) activity and altered the protein expression of TF in liver, lungs, heart and skin. In conclusion, the disturbances in redox status and hemostatic system induced by *B*. *jararaca* envenomation were modulated by rutin, suggesting it has a great potential to be used as an ancillary therapeutic agent for snakebites.

## Author summary

Snakebite is a neglected disease and a major health issue in tropical countries. Bites by *Bothrops* snakes (jararacas) are frequent throughout South and Central Americas and usually lead to inflammatory and bleeding manifestations in patients, which may be highly severe in some cases. Antivenom therapy is highly effective and the only officially recommended treatment for snakebites, but it is well known that it is not quite effective in blocking various secondary complications. We reasoned that rutin–an easily available and inexpensive plant-based compound–would have the ability to favorably minimize venom-induced low circulating fibrinogen levels, low blood platelet counts, local hemorrhage, bleedings, and raised production of reactive oxygen and nitrogen species during jararaca envenomation. We observed that rutin had remarkably beneficial effects, much higher than we originally expected, in most of the evaluated tests, and such effect was apparently not due to inhibition of the snake venom. Therefore, our results indicate that rutin has a great potential as an ancillary drug in concert with antivenom therapy to treat snakebites, particularly in countries where antivenom availability is scarce. Although we studied the effects of rutin on an experimental model, and further *in vivo* studies are necessary, our results are extraordinarily encouraging.

## Introduction

Worldwide, snakebites are considered a major public health issue, and, acknowledging that, the World Health Organization (WHO) declared snakebite envenomation as a neglected tropical disease. In Brazil, *Bothrops* snakes (lance-headed vipers) are responsible for approximately 20000 snakebites/year, and *Bothrops jararaca* is considered by WHO as a species of high medical importance since it causes numerous snakebites [1, 2].

*Bothrops jararaca* venom (BjV) is composed by a complex mixture of proteins, and most of them are grouped into the following families: snake venom metalloproteinases (SVMP), snake venom serine proteinases (SVSP), type-C lectins, phospholipases A_2_ (PLA_2_) and L-amino acid oxidases (LAAO) [3]. These proteins are responsible for the toxic activity of BjV, and evoke clinical disturbances in snakebite victims, such as intense inflammatory reactions (edema, local bleeding, and necrosis) at the site of the bite, and systemic bleeding (gingival bleeding, ecchymosis, petechiae, hematuria, epistaxis, and hemoptysis) [4]. Furthermore, envenomation induces thrombocytopenia, platelet dysfunction, consumptive coagulopathy, secondary fibrinolysis, a late mild fall in red blood cells (RBC) counts, and neutrophilic leukocytosis [5, 6].

A recent study of our group [6] showed that rats injected with BjV displayed increased tissue factor (TF, NCBI Reference Sequence: NP_001984.1) activity in plasma and tissues, and altered the protein expression of TF at the site of venom injection. TF is a 47-kDa transmembrane glycoprotein that initiates the extrinsic pathway of the coagulation cascade, and is present in platelets, monocytes, macrophages, endothelial cells, and microparticles of these cells. In normal physiological conditions, TF is found on its encrypted (inactive) form. However, under pro-inflammatory circumstances and/or injury, TF is decrypted and rapidly initiates blood coagulation *in vivo* [7]. Several mechanisms have been attributed to modulate TF function, and among them, the controversial regulatory activity of PDI, which is a thiol-isomerase and oxi-reductase chaperone that is required for thrombus formation *in vivo* and that has been reported to form an allosteric Cys_186_–Cys_209_ disulfide bond in TF, which is essential to its coagulating activity [7–9].

Envenomation by different genera of snakes–e.g., *Bothrops* [10], *Daboia* [11], *Echis* [12], and *Crotalus* [13]–have also been shown to evoke oxidative/nitrosative stress (ONS), characterized by an imbalance between the pro- and antioxidant systems. ONS may lead to extremely deleterious effects to living organisms [10] and it has been associated with several pathophysiological conditions, including hematological disturbances and inflammatory reactions resulting in leukocyte activation. In fact, increased levels of reactive oxygen and nitrogen species (ROS/RNS) may induce apoptotic signaling in RBC [14] and platelets [15], shortening their life span. During envenomation, the increase in reactive species has been attributed to both the ischemia-reperfusion tissue injury and the inflammatory reaction that occur following venom injection [10, 16]. Moreover, snake venom PLA_2_ and LAAO evoke lipid peroxidation and L-amino acid deamination, respectively, generating reactive species. Patients bitten by *B*. *jararaca* manifest ONS even one month after antivenom administration [10]. Although antivenom therapy is the recommended treatment for snakebites, it cannot directly block the secondary complications, such as reactive species generation, triggered directly or indirectly by venom toxins.

On that account, plant-based compounds are widely used in the treatment of several diseases, including those associated with ONS. Glycosides from quercetin group are remarkably abundant dietary flavonoids and display antioxidant action, such as metal ion chelation and reactive species scavenging, and block reactions and systems that generate reactive species [17]. Besides these actions, rutin (quercetin-3-rutinoside) binds to PDI, inhibiting its activity and, interestingly, the administration of rutin or isoquercetin has been shown to prevent thrombus formation in mice and humans [8, 18, 19]. However, if rutin administration also affects TF function or protein expression has never been evaluated.

Reasoning that the hemostatic disorders evoked by *B*. *jararaca* envenomation are multifactorial [6, 20], and that the concurrent imbalance in the redox status might be an additional mechanism to aggravate them, we hypothesized that ROS/RNS generation and TF encryption/decryption could be involved in the etiopathogenesis of hemostatic disorders during envenomation and might be mitigated by rutin administration. By using rutin, a potent antioxidant that controls thrombus growth and regulates ROS/RNS generation, we could address the link between generation of reactive species, TF decryption/encryption and hemostatic disturbances during *B*. *jararaca* envenomation; in addition, we investigated whether rutin could be used as a putative ancillary treatment to *B*. *jararaca* envenomation. We show herein that rutin has various beneficial effects during *B*. *jararaca* envenomation.

## Materials and methods

### Materials

Lyophilized venom from adult specimens of *B*. *jararaca* snakes was obtained from the Laboratory of Herpetology, Instituto Butantan. *Bothrops* antivenom was kindly donated by Instituto Butantan (lot: 1305077). Rutin was obtained from Sigma-Aldrich (USA). All other reagents were of analytical grade or better.

Antibodies: Anti-β-actin (A5316), anti-GADPH (G8795) and PDIa1 (P7372) were obtained from Sigma-Aldrich (USA). The anti-TF antibody (ab151748) was obtained from Abcam (USA). The anti-mouse IgG Alexa Fluor 488 (A11001) and anti-rabbit IgG Alexa Fluor 647 (A21245) were purchased from Thermo Fisher Scientific (USA).

### Ethics statement

Male Swiss mice, weighing 30–35 g, were obtained from the Animal Facility of Instituto Butantan, and were maintained with free access to food and water. The experimental procedures involving human donors, rats, and mice were in accordance with National Guidelines, and were approved, respectively, by the National Human Research Ethics Committee (Plataforma Brasil, CAAE: 51368615.5.0000.0065), and the Institutional Animal Care and Use Committee from Instituto Butantan (CEUAIB 4388061115) and the Faculdade de Medicina, Universidade de São Paulo (protocol 188/15). All procedures involving animals were in accordance with the National Guidelines of Conselho Nacional de Controle de Experimentação Animal (CONCEA) [21].

### Action of rutin on the major venom enzymes and on platelet aggregation *ex vivo*

To evaluate whether rutin directly inhibits SVMP, SVSP, LAAO and PLA_2_ present in BjV, the following substrates were used, respectively: azocoll, DL-BAPNA, L-leucine, and soybean lecithin [22]. The direct inhibition of the procoagulating activity of BjV by rutin was also tested in human and mouse plasmas, using the values of the minimum coagulant dose (MCD) for comparison [6]. Fixed concentrations of rutin (0.25 mg/mL for SVMP, SVSP and LAAO, and 1.25 mg/mL for PLA_2_) or two-fold serially diluted solutions (from 0.5625 to 9.0 mg/mL for MCD) of rutin were employed. BjV was two-fold serially diluted (from 0.0156 to 1 mg/mL for SVMP, SVSP, LAAO and MCD, or from 0.078 to 5.0 mg/mL for PLA_2_) and incubated with or without rutin for 30 min at 37°C. As positive controls for the inhibition of SVMP or SVSP, 13 mM Na_2_EDTA or 8 mM AEBSF, respectively, was incubated with BjV for 1 h at 37°C. Results were expressed as the percentage of inhibition of the enzymatic activities by rutin.

To test if BjV-induced platelet aggregation was inhibited by rutin, mouse blood (6 vol.) was collected from the caudal vena cava into ACD anticoagulant (1 vol.) (85 mM trisodium citrate, 71.4 mM citric acid, 111 mM dextrose, pH 6.2) and kept at 37°C. Thereafter 1 vol. of anticoagulated blood was gently homogenized with 2 vol. of washing solution [6 vol. of Dulbecco’s Modified Eagle’s Medium (DMEM, pH 7.4) to 1 vol of ACD], laid onto 1 mL of Histopaque 1077 (Sigma-Aldrich, USA), and centrifuged at 700 *g* for 30 min at room temperature. The upper layer was added up to 2 mL of DMEM/ACD containing prostaglandin E_1_ (0.5 μL, 200 μg/mL) and centrifuged at 9500 *g* for 90 s at room temperature. Supernatants were discarded and the pellet of platelets was resuspended in DMEM/ACD/PGE_1_. Centrifugation and resuspension of platelets were carried out 2 more times and finally platelets were resuspended in DMEM, and platelet count was adjusted to 5 x 10^5^ platelets/mL. Washed platelets were stimulated by BjV alone or BjV+rutin (final concentrations: BjV, 24.4 μg/mL and rutin, 220 μg/mL). Platelets were also pre-incubated with rutin for 15 min at 37°C and then BjV was added. Platelet aggregation was recorded for 5 min using a Chrono-log aggregometer (USA) [22].

### Venom and rutin solutions

BjV was dissolved in sterile saline (0.8 mg/mL); rutin (7.2 mg/mL) was dissolved in a solution containing 1 vol. of propylene glycol and 1 vol. of saline. The solutions were prepared immediately before use. The dose of BjV (1.6 mg/kg b.w., s.c. route) was selected based on previous tests to determine a dose that evoked hemostatic disturbances in mice similar to those observed in rats [6, 23] and humans [5]. The dose of rutin (14.4 mg/kg b.w, s.c. route) was established based on previous experiments, showing that this dose was effective to modulate redox status and hemostatic parameters.

### Experimental groups and procedures

**Fig 1:**
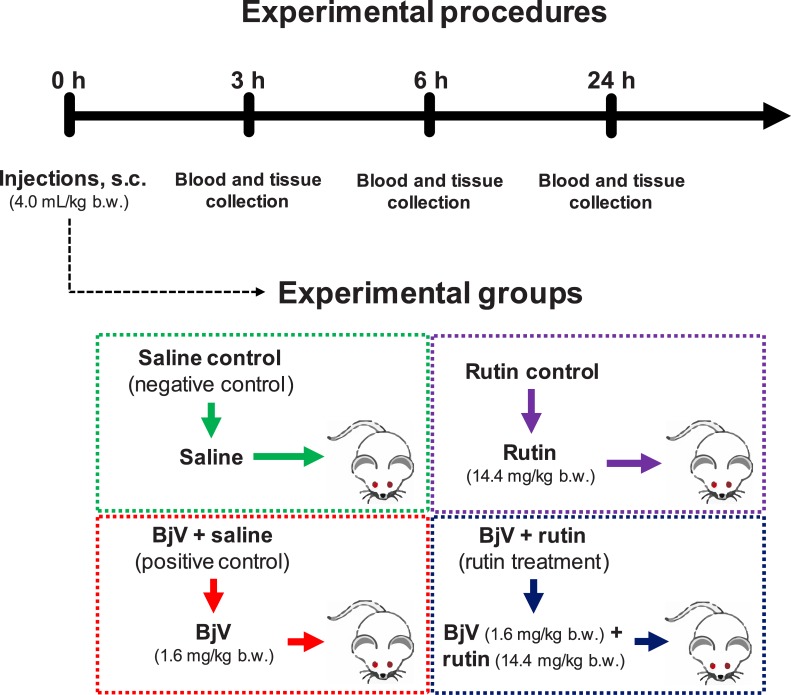
Schematic representation of experimental groups, and the timing of injections and sample collections.

Animals were randomly allocated in four experimental groups (n = 6/group/time interval) (Fig 1) and received (4.0 mL/kg b.w., s.c.): *saline (*saline control, i.e., the negative control); *rutin* (rutin control, 14.4 mg/kg b.w. of rutin); *BjV+saline* (positive control, 1.6 mg/kg b.w. of BjV); and *BjV+rutin* (rutin treatment, 14.4 mg/kg b.w. of rutin and 1.6 mg/kg b.w. of BjV). The solutions were incubated at 37°C for 30 min before administration.

At 3, 6 and 24 h after injection, animals (5–6 mice/group) were anesthetized with isoflurane (induction and maintenance at 2.5%), and blood and tissues were collected (Fig 1). Blood was collected from the caudal vena cava into plastic syringes to obtain complete blood cell counts (CBC), blood smears and plasma. Blood was added to flasks containing the following anticoagulants: CTAD (75 mM trisodium citrate, 42 mM citric acid, 139 mM dextrose, 15 mM theophylline, 3.7 mM adenosine, 0.2 mM dipyridamole, and 2 μM imipramine), 3.2% trisodium citrate, or 269 mM Na_2_EDTA. *Bothrops* antivenom (1 vol. to 100 vol. of blood) was added to all flasks to avoid *in vitro* clotting of the blood induced by BjV. Fragments of liver, lungs, heart (50–100 mg) and skin (0.5–0.75 cm^2^ around the site of s.c. injection) were collected in flasks containing RIPA buffer (50 mM Tris-HCl, 150 mM NaCl, 1% Triton X-100, 1% sodium deoxycholate, 0.1% SDS, 2 mM Na_2_-EDTA, and a protease inhibitor cocktail, pH 7.5) or HBSA buffer (20 mM HEPES-NaOH buffer, pH 7.5, containing 100 mM NaCl, 0.02% NaN_3_ and 1 mg/mL bovine albumin), both containing *Bothrops* antivenom, and frozen at -80°C. Tissues were disrupted using FastPrep-24 (#6004–500, MP Biomedicals) for 60 s at 6.5 m/s, followed by 3 cycles of freezing in dry ice and thawing in a water bath at 37°C. Finally, samples were centrifuged at 13000 *g* for 10 min at 4°C, and supernatants were collected and frozen at -80°C. Protein concentration in the supernatants was determined by bicinchoninic acid assay [24]. As reference samples, pools of normal plasma and each organ (liver, lungs, heart and skin) were obtained and assayed on each determination.

### Redox status parameters

The measurement of reactive species levels in plasma was assayed based on previous reports [25, 26] by detecting the fluorescence of DCFH-DA. The results were analyzed using a DCF standard curve (from 0.097 to 6.250 nM DCF). The total antioxidant capacity (TAC) was determined based on the CUPRAC colorimetric assay [27]. As a standard curve, 2-fold serially dilutions of reduced L-glutathione (from 12.5 to 300 μM) were used. Results obtained in each assay were normalized using a normal mouse plasma pool as reference, and were expressed in arbitrary units.

### CBC and hematological assays

CBC was performed in an automated cell counter BC-2800 Vet (Mindray, China), and differential counts were carried out in blood smears stained panchromatically. Levels of plasma hemoglobin were measured by the peroxidase method [28]. The results were quantified based on a standard curve of hemoglobin (from 3.125 to 200 μg/mL) and normalized using a reference plasma pool as described above on DCF method. Plasma fibrinogen was analyzed using a colorimetric assay [29]. Prothrombin time (DiaPlastin, DiaMed, Brazil), TF activity (Actichrome TF Kit, Sekisui, USA), and TF antigen (mouse-TF ELISA kit, Elabscience, USA) were assayed in plasma according to manufacture instructions.

### Tail bleeding and local hemorrhage assays

Both tail bleeding and local hemorrhage were assessed in the same mice (5–11 mice/group). The tail bleeding assay was used to evaluate hemostasis *in vivo*, and was evaluated by determining the volume of blood (hemoglobin) leaked after tail amputation; the hemorrhagic activity of venom was evaluated by measuring the area and color intensity of hemorrhage at the subcutaneous region surrounding the site of injection [30, 31]. Briefly, animals received the treatments s.c. at the dorsum, and after 3 h they were anesthetized and maintained under isoflurane anesthesia during the whole procedure of tail bleeding. A distal 10-mm segment of the tail was amputated and the tail was immediately immersed in 50 mL of isotonic solution (154 mM NaCl, 2 mM CaCl_2_) at 37°C for 15 min. Thereafter, mice were euthanized, and the suspension was centrifuged at 1900 *g* for 15 min at room temperature. The pelleted blood cells were resuspended in 1 mL of saline, homogenized, and 100 μL of this mixture were added to 5 mL of von Kampen-Zijlstra reagent (200 mg K_3_[Fe(CN)_6_], 50 mg KCN, 120 mg KH_2_PO_4_, 50 mg NaCl and 1 mL Triton X-100 per liter). The suspension was read at 540 nm and the results were expressed as mg of hemoglobin per sample [32]. The analysis of the area and intensity of hemorrhage was carried out as described elsewhere [33], using the images of the subcutaneous of mouse skin captured with ImageScanner III (GE Healthcare, USA), and the results were expressed as hemorrhagic units.

### TF protein expression and activity

Tissue supernatants were electrophoresed in 10% SDS-PAGE gels and blotted to evaluate protein expression of TF, PDI, β-actin and GADPH. Nitrocellulose membranes were blocked with 5% nonfat dry milk, and thereafter incubated with primary antibodies anti-TF, anti-β-actin and anti-GAPDH (1:5000), and later on with secondary antibodies (anti-mouse IgG conjugated with Alexa Fluor 488 and anti-rabbit IgG conjugated with Alexa Fluor 647, 1:5000). Fluorescence emission was captured in a ChemiDoc MP system (Bio-Rad, USA), and images were analyzed using ImageLab software (version 5.2.1, Bio-Rad). Relative quantification was performed as described elsewhere [6, 34], with normalization based on the values of reference pools for each organ.

TF activity in tissue samples was measured by a coagulant assay [35] using organ fragments immersed in HBSA. Tissue supernatants, obtained as mentioned previously, were incubated with a pool of normal plasma (10% rat, 90% human) and after adding 25 mM CaCl_2_, the clotting time was measured in a Start4 coagulometer (Stago, France). Once mouse TF is known to be less efficient to activate human factor VII/VIIa, and the volume of plasma required for clotting assays is relatively high, the mixture of plasma pools from normal rats and humans [36–38] was used as a surrogate to assay mouse TF in tissue samples. TF activity was calculated based on a standard curve of commercial thromboplastin (DiaMed, Brazil), and was normalized using the reference sample of tissues. Results were also used to calculate the TF activity/ TF protein expression ratio.

### Statistical analyses

The *a priori* calculation of sample size (6 mice per group) was determined in G*Power 3 software (http://www.gpower.hhu.de), taking a two-way ANOVA design, a β error of 20% (power 0.8), an α error of 0.05, and an effect size of 0.2 (based on previous data about platelet count and fibrinogen levels in BjV-injected animals and controls). Normal distribution and homoscedasticity of the results were analyzed using the software STATA^TM^, version 10, and data were transformed whenever necessary. Depending on the statistical analysis, one-way, two-way ANOVA, or Kruskal-Wallis test was used, followed by *post-hoc* tests (Bonferroni, Student-Newman-Keuls or Dunn’s tests). The softwares SPSS (version 22) and SigmaPlot (version 12.0) were employed for these analyses. *Post-hoc* power analysis of ANOVA’s were greater than 0.87. SPSS was also used to determine the Pearson correlation coefficients and ROC curves between two variables. Results were considered statistically significant when p< 0.05, and the data were expressed as mean ± standard error of mean (s.e.m.). Statistical tests used for each variable were described in figure legends.

## Results

### Rutin does not inhibit BjV enzymes *in vitro* nor BjV-induced platelet aggregation *ex vivo*

**Fig 2:**
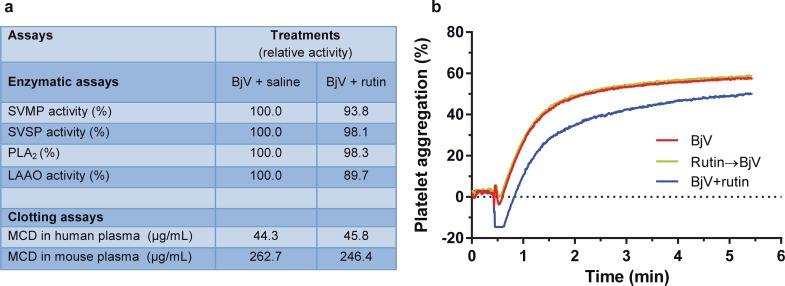
*In vitro* activity of rutin on *Bothrops jararaca* snake venom. (**a**) Relative enzymatic activities and MCD values (clotting activity) of *B*. *jararaca* venom (BjV) following incubation with rutin. BjV was incubated with rutin (BjV+rutin) or saline (BjV+saline) and was tested for inhibition of: snake venom metalloproteinases (SVMP), snake venom serine proteinases (SVSP), phospholipases A_2_ (PLA_2_) and L-amino acid oxidases (LAAO) (for details see Material and Methods). BjV incubated with Na_2_EDTA and AEBSF showed 14.0% and 1.6% of the original activity, respectively. The values of MCDs of BjV incubated with Na_2_EDTA and AEBSF in human plasma were 137.8 and 102.5 μg/mL, respectively. The clotting activity of mouse plasma induced by BjV was abrogated by incubation with Na_2_EDTA, and the MCD of BjV incubated with AEBSF was 642.3 μg/mL. (**b**) Platelet aggregation tracings of washed mouse platelets (5 x 10^5^ platelets/mL) stimulated by BjV alone, BjV+rutin or platelets pre-incubated with rutin for 15 min at 37°C and then stimulated by BjV (Rutin→ BjV). Data were expressed as percentage of platelet aggregation.

Rutin minimally interfered in the activity of SVMP, SVSP, PLA_2_ and LAAO. Furthermore, the clotting activity of BjV–represented by MCD values in human and mouse plasmas–was similar in the presence or absence of rutin (Fig 2A). As positive controls, Na_2_EDTA, a SVMP inhibitor, inhibited 86% of the collagenolytic activity of SVMP, completely inhibited the clotting activity of BjV in mouse plasma and diminished the clotting activity of BjV by 3-fold in human plasma; on the other hand, AEBSF, an inhibitor of SVSP, decreased the catalytic activity of SVSP by 98.4%, and also blocked the coagulant capacity of BjV by 2-fold in human and mouse plasma. These data showed that rutin failed to inhibit SVMP, SVSP, PLA_2_ and LAAO *in vitro*, as well as the coagulant activities of BjV. The inhibitory activity of rutin on BjV-induced platelet aggregation was also tested *ex vivo*, showing that when rutin was incubated with washed platelet suspensions for 15 min and later stimulated with BjV, the same extent of platelet aggregation induced by BjV alone (approximately 60%) was observed (Fig 2B). On the other hand, if BjV was incubated with rutin for 30 min, and both were added simultaneously to the platelet suspensions, platelet shape change was more prolonged, leading to a mild reduction in the extent of platelet aggregation (≈ 50%). These results indicated that rutin possibly bound to components in BjV important to induce platelet activation, however this fragile binding was rapidly broken in contact with platelets, so that platelet activation and aggregation occurred normally.

### Envenomation induces redox status imbalance and rutin controls reactive species levels

**Fig 3:**
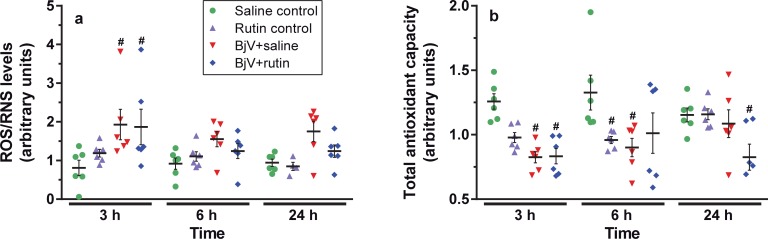
Plasma levels of ROS/RNS (**a**) and total antioxidant capacity (**b**) in mice at 3, 6 and 24 h after treatments. Two-way ANOVA was used, followed by Bonferroni *post-hoc* test; # p< 0.05 and # # p< 0.001 when compared to saline control group on the respective time; * p< 0.05 and ** p< 0.001 when compared to BjV+saline group on the respective time. Data were expressed as mean ± S.E.M. (n = 4-6/group).

As shown in Fig 3A for the DCF assay, in comparison with the saline group, a marked increase in ROS/RNS levels was observed following BjV injection from 3 to 24 h, but it was more evident at 3 h (p< 0.05). Rutin tended to limit the rise in ROS/RNS levels in animals treated with BjV, particularly at 6 and 24 h, but the differences among BjV+saline and BjV+rutin groups were not statistically significant (p = 0.178 at 6 h and p = 0.238 at 24 h). Concomitantly, but not in direct parallel with the rise in ROS/RNS levels, there was a statistically significant drop in TAC levels (Fig 3B) in mice injected with BjV+saline and BjV+rutin at 3 h (p<0.05). Although TAC and DCF plasma levels varied inversely in the experimental groups, only a modest correlation was noticed (r = -0.245, p = 0.0399, n = 71), evidencing that they evaluated different aspects of ONS. Rutin failed to restore TAC levels in the BjV+rutin group, even though it decreased ROS/RNS levels. Altogether, these data showed that BjV generated reactive species, particularly hydroxyl and peroxynitrite radicals detected by DCF [39], and decreased TAC levels. The reduction in TAC levels reinforced the idea that low non-enzymatic antioxidant compounds have been consumed during envenomation. Rutin partially controlled the generation of reactive species, but rutin alone did diminish TAC levels *in vivo*.

### Envenomation decreases RBC values: Protection by rutin

**Fig 4:**
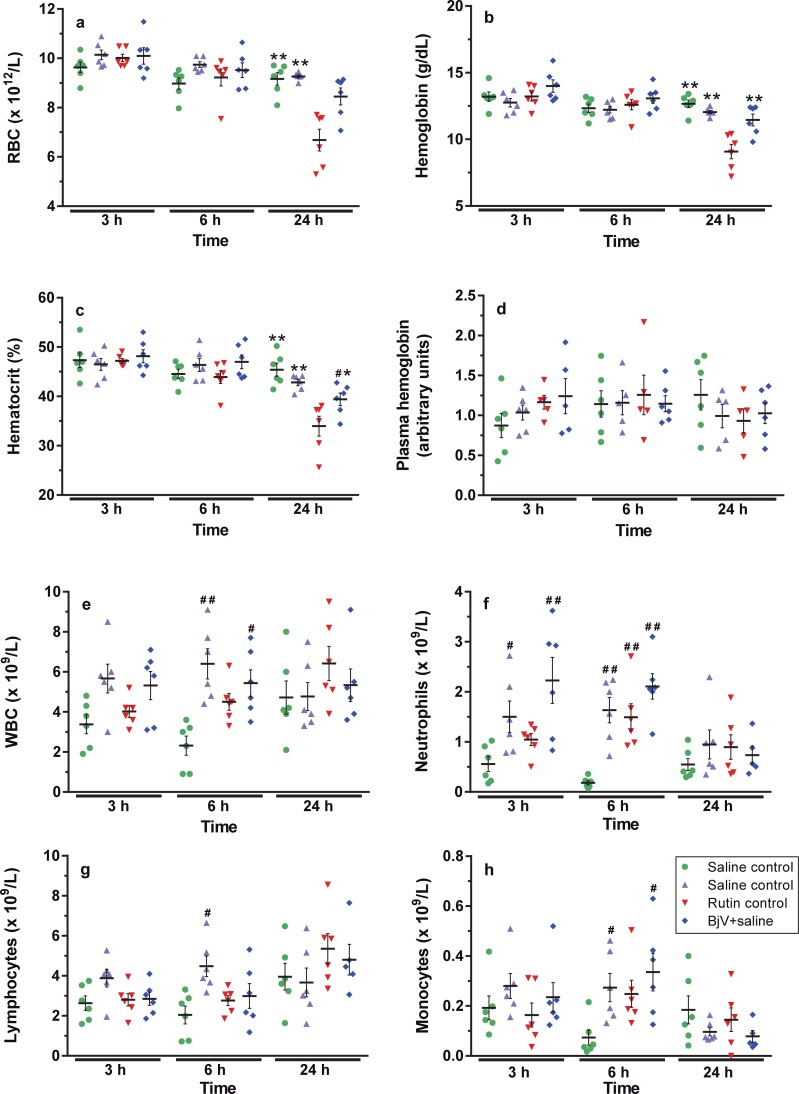
RBC counts (**a**), hemoglobin (**b**), hematocrit (**c**), plasma hemoglobin (**d**), WBC counts (**e**), and absolute counts of lymphocytes (**f**), neutrophils (**g**) and monocytes (**h**) in mice at 3, 6 and 24 h after treatments. Two-way ANOVA was used, followed by Bonferroni *post-hoc* test, except for plasma hemoglobin (Kruskal-Wallis test); # p< 0.05 and # # p< 0.001 when compared to saline control group on the respective time; * p< 0.05 and ** p< 0.001 when compared to BjV+saline group on the respective time. Data were expressed as mean ± S.E.M. (n = 4-6/group).

Erythron values did not vary importantly among groups, except at 24 h when values of RBC (Fig 4A), hematocrit (Fig 4B) and hemoglobin (Fig 4C) decreased around 25–30% in BjV+saline group compared to all control groups (p< 0.001). Importantly, rutin prevented this fall at 24 h, and the BjV+rutin group showed RBC values similar to those of controls. The morphological analysis of RBC in blood smears showed that half of the animals in the BjV+saline group showed anisocytosis and polychromasia at 24 h, whereas they were not observed in the BjV+rutin group. We also analyzed plasma hemoglobin levels to verify if the decrease in RBC parameters was due to intravascular hemolysis. As shown in Fig 4D no statistically significant difference among groups was noticed at any time period, indicating that BjV evoked RBC disturbances, and that rutin could successfully prevent them.

An increase in WBC and neutrophil counts (p< 0.001) was noticed in the BjV+saline group at 6 h (Fig 4E and 4F). Mice that received rutin showed higher WBC, neutrophil, and monocyte counts (Fig 4E, 4F and 4H) in comparison with the saline group at 3 and 6 h (p< 0.05). Moreover, in the control rutin group at 6 h, lymphocyte counts (Fig 4G) were also elevated (p< 0.05).

### Rutin prevents BjV-induced coagulopathy, but not thrombocytopenia

**Fig 5:**
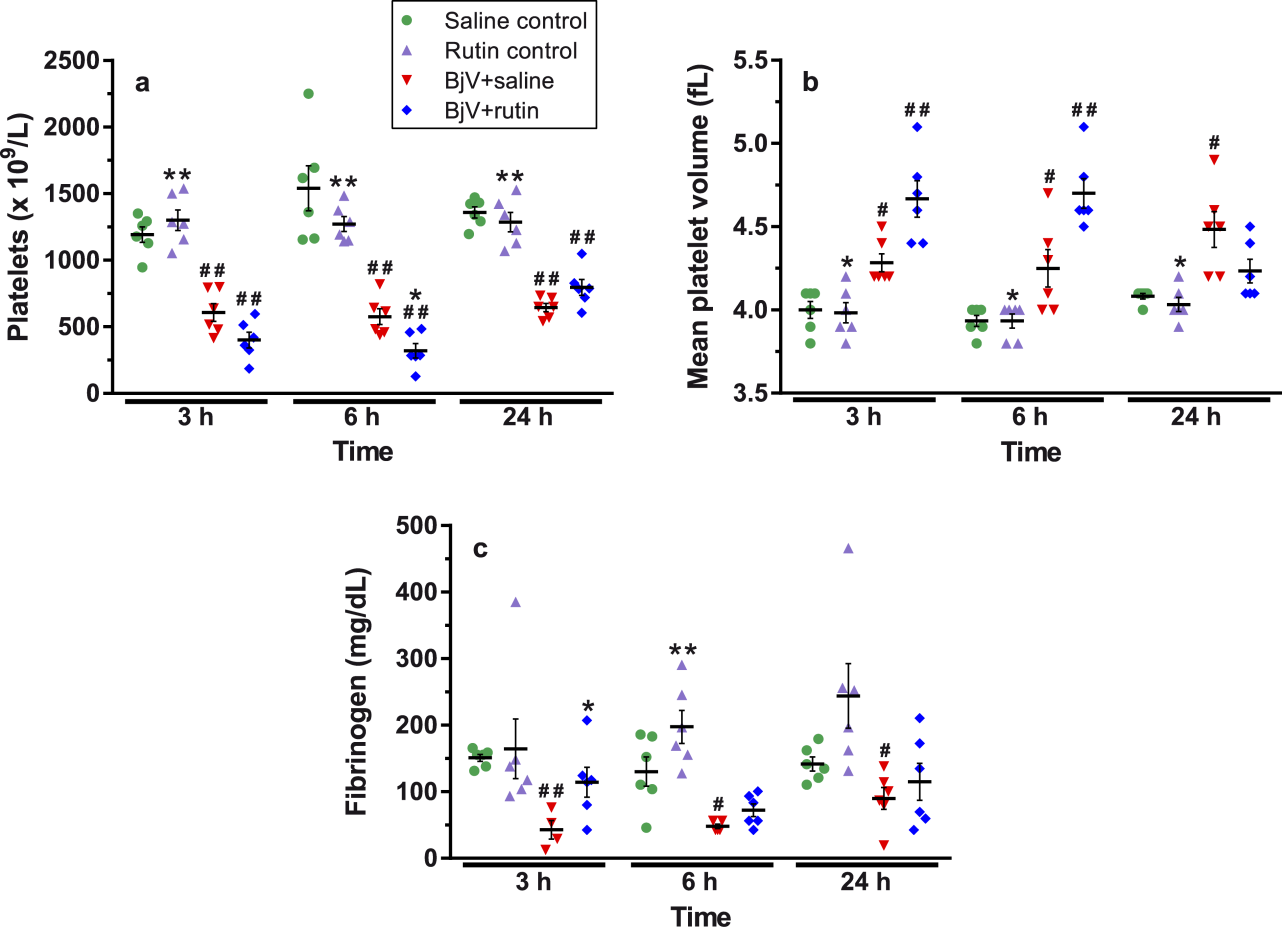
Platelets counts (**a**), mean platelet volume (**b**), plasma fibrinogen levels (**c**) at 3, 6 and 24 h after treatments. Two-way ANOVA were used, followed by Bonferroni *post-hoc* test; # p< 0.05 and # # p< 0.001 when compared to saline control group on the respective time; * p< 0.05 and ** p< 0.001 when compared to BjV+saline group on the respective time. Data were expressed as mean ± S.E.M. (n = 4-6/group).

BjV (alone or with rutin) markedly decreased platelet counts (Fig 5A) in comparison with the saline control (p< 0.001) at all time periods. Simultaneous to the fall in platelet counts, an increase was noticed in the mean platelet volume (MVP, Fig 5B) over time (p< 0.05), showing that the consumption of circulating platelets was followed by the release of new larger platelets into the blood stream.

Pearson correlation coefficients and ROC curves between DCF or TAC, and hematological and biochemistry data evidenced that only platelet counts showed a statistically significant correlation with TAC (r = 0.429, p = 2.48 × 10^−5^, n = 72) or DCF (r = -0.352, p = 2.59 × 10^−3^, n = 71), implying that thrombocytopenia was mildly associated with ONS. In fact, the values of the area under the curve (AUC) for ROC curves between DCF (0.728 ± 0.063; cutoff 1.3) or TAC (0.618 ± 0.072; cutoff 1.1) and platelet counts were modestly high, and thereby they could not be considered good discriminators for the development of thrombocytopenia.

BjV induced a marked drop in fibrinogen levels (Fig 5C), which tended to recover at 24 h (p< 0.05). Interestingly, rutin prevented animals from fibrinogen consumption during envenomation, once no statistically significant differences were noticed between the BjV+rutin and saline groups over time (p = 0.935 at 3 h, p = 0.233 at 6 h, and p = 1.000 at 24 h). Once rutin blocked the drop in fibrinogen levels induced by BjV occurring as soon as 3 h after injection, we explored if rutin could attenuate other major hemostatic disturbances induced by BjV at this time period. No statistically significant correlation was noticed between DCF or TAC levels, and fibrinogen levels.

### BjV induces TF activation in the acute phase of envenomation

**Fig 6:**
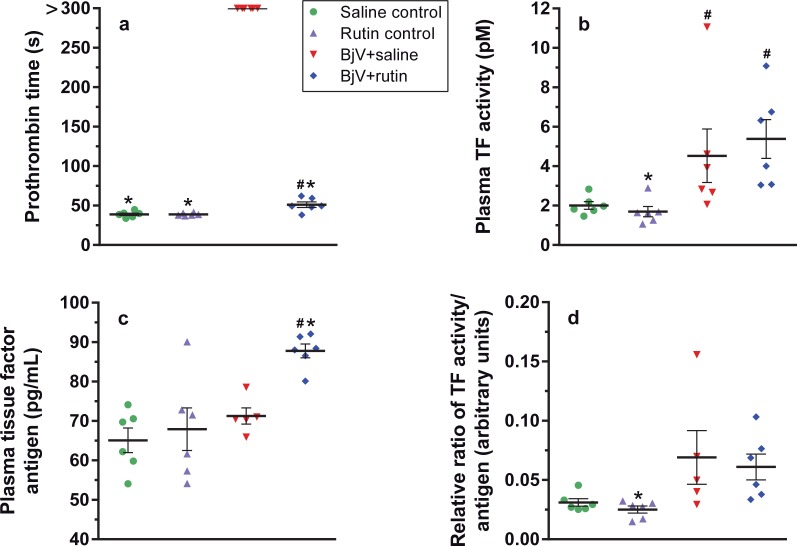
Prothrombin time (**a**), plasma TF activity (**b**), plasma TF antigen (**c**), ratio of TF activity/TF antigen (**d**) in mice at 3 h after treatments. One-way ANOVA was used for TF antigen and ratio, followed by Bonferroni *post-hoc* test; Kruskal-Wallis test was used for TF activity and prothrombin time, followed by Student-Newman-Keuls *post-hoc* test; # p< 0.05 and # # p< 0.001 when compared to saline control group on the respective time; * p< 0.05 and ** p< 0.001 when compared to BjV+saline group on the respective time. Data were expressed as mean ± S.E.M. (n = 5-6/group).

Considering the hemostatic disturbances evoked by BjV at 3 h after the envenomation, we investigated what was occurring to the extrinsic pathway of the coagulation cascade at this time period. As expected, 3 h after envenomation the BjV+saline group showed a prolongation of prothrombin time (>300 s) (p< 0.05), and despite the difference noticed between the BjV+rutin and saline control groups (p< 0.05), rutin markedly shortened prothrombin time in comparison with BjV+saline group (Fig 6A). Furthermore, increased TF activity (p< 0.05) (Fig 6B) and unaltered TF antigen levels (p = 1.000, Fig 6C) were noticed in the BjV+saline group, leading to a tendency towards the increase in TF activity/antigen ratio (Fig 6D). On the other hand, the BjV+rutin group displayed an increase in both the activity and antigen levels of TF (p< 0.05), showing thereby a lower TF activity/antigen ratio compared with the BjV+saline group. Thus, these results indicate that envenomation triggered the coagulation cascade inducing not only the direct activation of coagulation factors, but also TF decryption, and that both factors possibly contribute to the consumptive coagulopathy. Rutin in turn failed to decrease TF activity in plasma, but, on the contrary, did augment the levels of TF antigen in envenomed animals. These findings demonstrated that rutin did not interfere in TF decryption, and favored the increase in TF levels in plasma. Nonetheless, and most importantly, rutin markedly reduced prothrombin time, suggesting that its beneficial activity was not associated with the modulatory activity of TF encryption/decryption.

### Rutin restores hemostasis and avoids local hemorrhage

**Fig 7:**
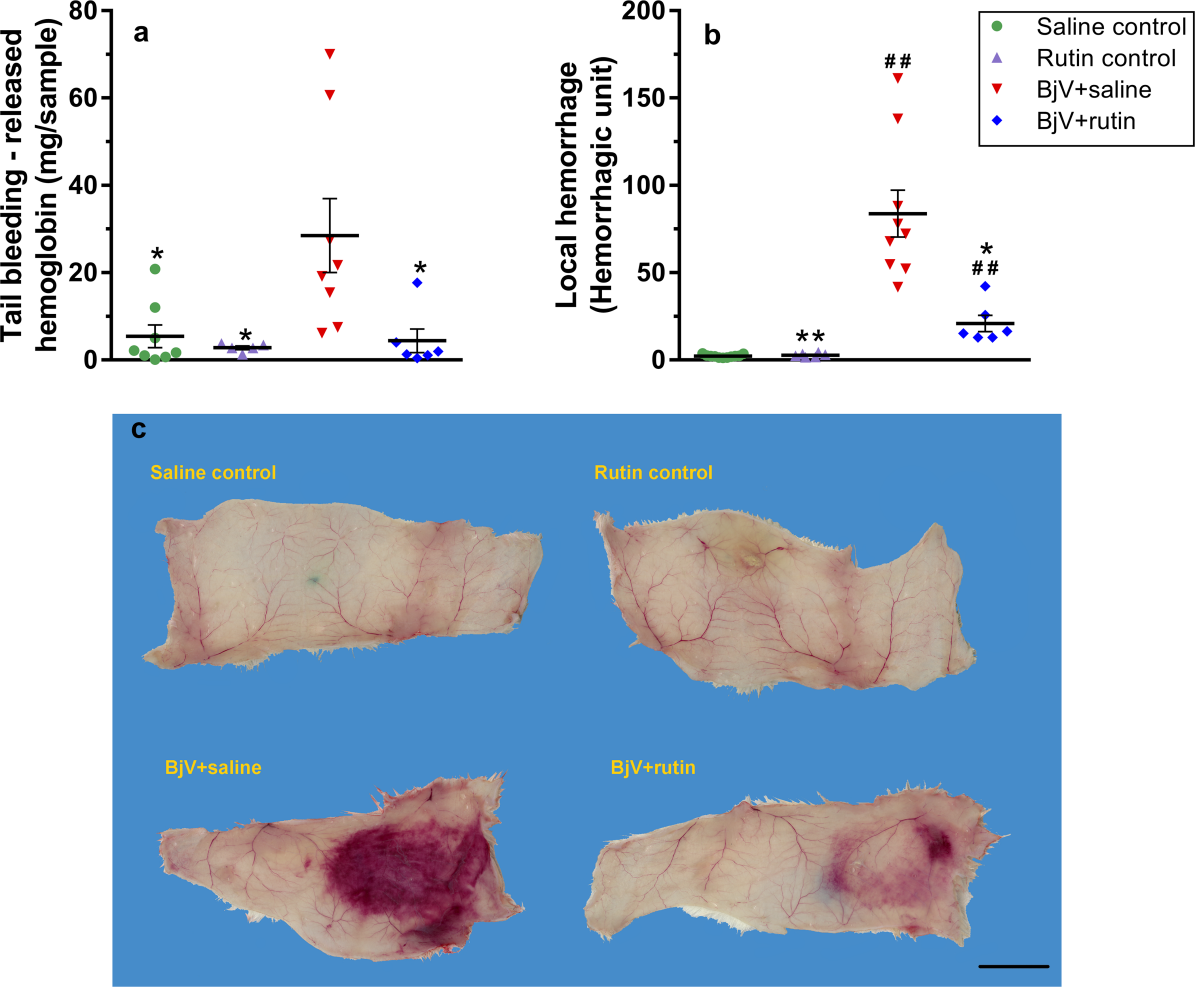
Tail bleeding (**a**) and local hemorrhage (**b**) in mice at 3 after treatments. One way ANOVA was used, followed by Bonferroni *post-hoc* test; # p< 0.05 and # # p< 0.001 when compared to saline control group on the respective time; * p< 0.05 and ** p< 0.001 when compared to BjV+saline group on the respective time. Data were expressed as mean ± S.E.M. (n = 5-11/group). Representative images of subcutaneous hemorrhage (**c**), used to calculate local hemorrhage. Bar size: 2 cm.

The changes induced by BjV in blood coagulation and platelets were also echoed by increased tail bleeding (Fig 7A), a test that evaluates hemostasis *in vivo*. On the other hand, the BjV+rutin group showed a marked reduction in tail bleeding–which was not statistically different from the saline control group (p = 1.000)–showing that rutin was able to circumvent hemostatic alterations induced by BjV. Rutin alone did not interfere in tail bleeding. Furthermore, the well characterized local hemorrhage induced by BjV at the site of venom injection (Fig 7B and 7C) was also remarkably attenuated by rutin (p< 0.05 when compared to BjV+saline). These findings imply that rutin has *in vivo* actions that counteract the toxic activities of BjV.

### Rutin modulates TF expression in organs

**Fig 8:**
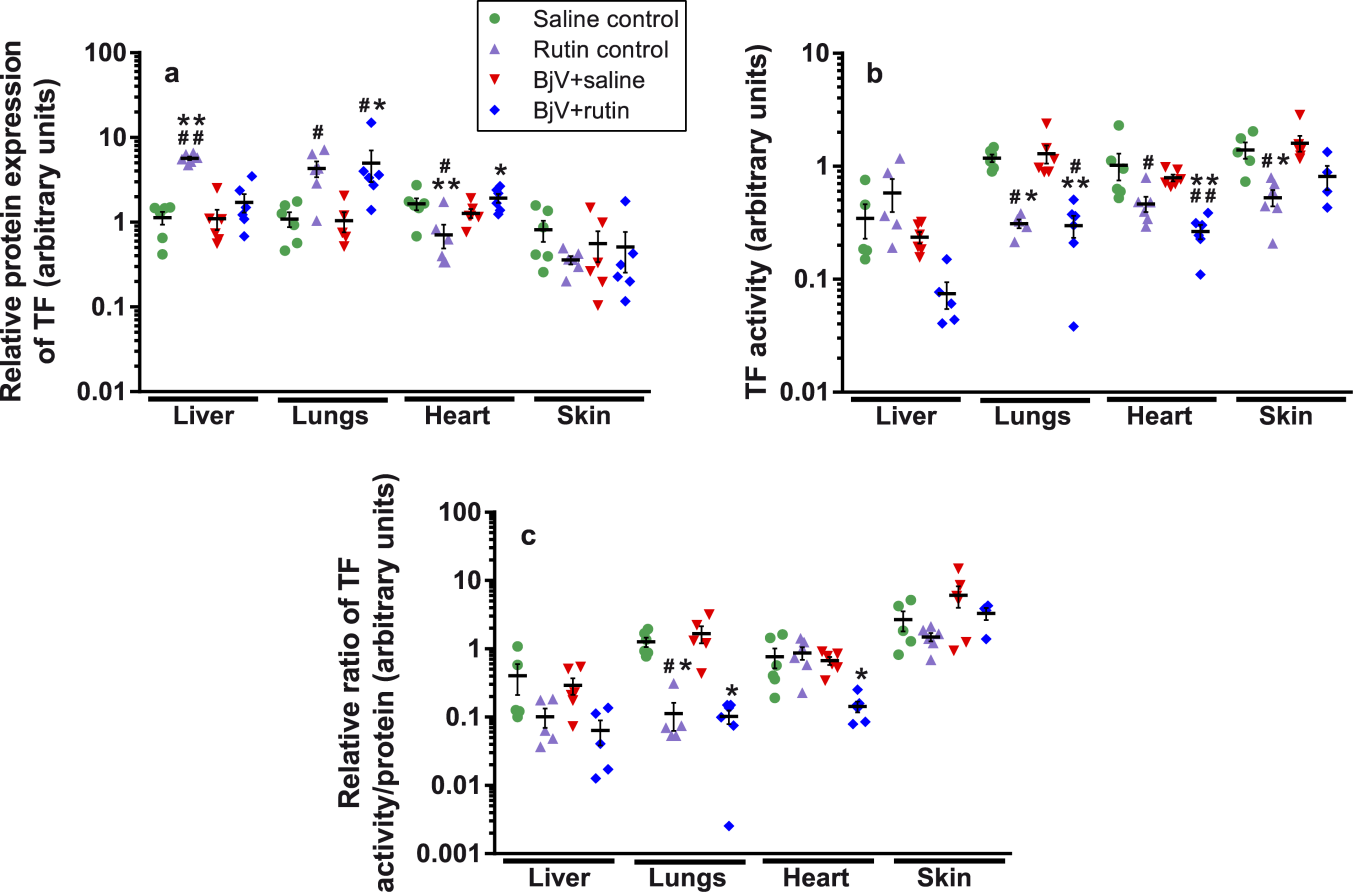
Protein expression of TF (**a**), TF activity (**b**) and ratio of TF activity/TF protein expression (**c**) in liver, lungs, heart and skin in mice at 24 h after treatments. One-way ANOVA was used, followed by Bonferroni *post-hoc* test (protein expression and activity of TF), or Kruskal-Wallis test was used, followed by Dunn’s *post-hoc* test (TF ratio); # p< 0.05 and # # p< 0.001 when compared to saline control group; * p< 0.05 and ** p< 0.001 when compared to BjV+saline group. Data were expressed as mean ± S.E.M. (n = 4-6/group).

Protein expression of endogenous proteins (GAPDH and β-actin, supporting information, S1 Fig) and TF (Fig 8A) was analyzed in samples of liver, lungs, heart, and skin (at the site of venom injection) at 24 h, and the profile of protein expression changed depending on the organ. BjV failed to evoke statistically significant differences in TF, GAPDH and β-actin expression in regard to the saline control in most organs, except for decreasing GAPDH expression in the skin. However, most of protein expression changes were induced by rutin alone, which upregulated or downregulated all studied proteins systemically. The same profile of changes was obtained for PDI, supporting information, S2 Fig). In the BjV+rutin group, protein expression was similar to that of the BjV+saline group in the liver, whereas in the lungs and skin it was similar the rutin control. Only in the heart, protein expression behaved differently from the BjV+saline or rutin control groups. These results evidence that BjV exerted little influence on protein expression of TF at 24 h. On the other hand, surprisingly rutin alone induced a drastic change in protein expression, from either endogenous proteins (GAPDH and β-actin) or TF.

### Rutin decreases TF activity in tissues

In order to investigate if rutin could alter TF decryption systemically, TF activity was also assayed in organs at 24 h. In addition, TF activity/protein expression ratio was also calculated using both the TF activity and TF protein expression values reported above (Fig 8B and 8C). As noticed for protein expression results, no alterations were observed in TF activity and TF activity/protein expression ratio in all organs in the BjV+saline group. However, animals injected with rutin (rutin control and BjV+rutin groups) showed lower TF activity (p< 0.05) in the lungs, heart and skin when compared to the saline control or BjV+saline groups (Fig 8B). Interestingly, in the rutin control group the TF activity/protein ratio (Fig 8C) decreased significantly only in the lungs (p< 0.05) in comparison with the saline control and BjV+saline groups, although the same paradigm was also detected in the liver and skin. The BjV+rutin group had decreased TF activity/antigen ratio in the lungs and heart when compared to the BjV+saline group (p< 0.05).

## Discussion

*Bothrops jararaca* venom displays pro-inflammatory, hemorrhagic and anti-hemostatic activities [22] that elicit clinical manifestations in patients bitten by *B*. *jararaca* snakes. However, the mechanisms of action whereby clinical manifestations develop are not completely understood, as well as why some patients develop complications resulting from severe hemostatic disturbances [40] and from ONS [10]. Herein we scrutinized the complex relationship between ONS and hemostatic disturbances, mainly related to TF, and the potential of a natural antioxidant, rutin, to be used as an ancillary treatment to snakebites.

Firstly, we investigated the effect of rutin on BjV, since both were pre-incubated prior to injection in mice. Rutin failed to directly inhibit the main protein families tested *in vitro*, indicating that it modulates the very pathophysiological events evoked by snake envenomation. BjV increased the levels of ROS/RNS and decreased the levels of antioxidants, which are features of redox status imbalance, in mice at least for 24 h, which are in agreement with previous reports [41, 42]. Recently, ONS has been considered critical for the pathophysiology of snake envenomation [43–45], and in one prospective study in human patients bitten by *Bothrops* snakes [10] the levels of oxidative stress markers were altered for long periods of time. Once the presence of ONS is an incipient observation in *B*. *jararaca* snake envenomation, the coexistence of the redox status imbalance and the development of hemostatic disturbances led us to wonder whether the former could be interfering or be associated with the latter, as reported elsewhere for other genus of snakes [11, 12].

In mice, BjV induced various hemostatic and hematological disturbances. Thrombocytopenia is a characteristic manifestation in *B*. *jararaca* envenomation, and it is not due to the action of SVMP or SVSP, nor evoked by platelet consumption at the sites of hemorrhagic spots; moreover it is not directly associated with fibrinogen consumption [6, 23]. Although rutin inhibits platelet aggregation induced by physiological agonists [17, 18], our results evidence that rutin failed to directly inhibit BjV-induced platelet aggregation and to protect animals from platelet consumption. Additionally, even when redox status parameters were unaltered during envenomation, thrombocytopenia could be observed, indicating that thrombocytopenia is not directly caused by ONS during envenomation.

On the contrary, BjV-induced coagulopathy–characterized by fibrinogen consumption, secondary fibrinolysis, and moderate falls in levels of factor II and X [6, 23]–was drastically reduced by the use of rutin. Fibrinogen consumption is considered the primary cause of prolonged coagulation time in *B*. *jararaca* envenomation [6], and herein rutin prevented BjV-induced coagulopathy. In addition, SVMP-induced intravascular thrombin generation—by factor II and X activators—has a pivotal role in causing fibrinogen consumption during *B*. *jararaca* envenomation in rats [6, 23].

Our results confirmed that BjV induced an increase in TF activity in plasma in the acute phase of envenomation, possibly triggering the coagulation cascade and contributing thereby to the hemostatic disturbances. Interestingly, the alteration in TF in plasma was not due to an increase in TF antigen concentration, but to an increase in TF activity, suggesting TF decryption. Coagulant and non-hemorrhagic SVMP [46, 47] might have a role in increasing TF by stimulating monocytes and endothelial cells, and in fact a coagulant SVMP has been shown to modulate TF activity in mononuclear cells *in vivo* [48]. Thus, our data demonstrate that the raise in circulating levels of TF activity is a remarkable response to BjV-induced systemic injury, possibly leading to true disseminated intravascular coagulation. However, even though there are snake venom enzymes that cause direct consumption coagulopathy, the contribution of TF activation has yet to be determined. Nonetheless, TF activation may be important to hemostatic complications resulting from snake envenomation, as recurrent coagulopathy and thrombocytopenia following antivenom therapy do occur, and may be due not exclusively to low doses of antivenom used in the treatment. It is however still unclear which mechanism is responsible for the activation of TF during envenomation and how important it is to consumptive coagulopathy. Considering that *Bothrops* snake venoms and their coagulant SVMP can up-regulate TF activity *in vitro* and *in vivo* [6, 46, 48], and that rutin prevented coagulopathy, we investigated whether rutin was controlling TF activity. Our initial rationale by choosing rutin was its properties as an antioxidant, a direct inhibitor of thrombin [49], and an antithrombotic drug that prevents thrombus formation *in vivo* [8, 18, 19] by inhibiting extracellular and membrane-anchored PDI, an enzyme that modulates TF activity. Rutin administration failed to decrease TF activity in plasma, indicating that the amelioration induced by rutin in the coagulopathy of the envenomation is not due to a direct interference in the TF activity in blood stream. Furthermore, it was essential to elucidate whether BjV altered not only blood parameters, but also TF at the site of venom injection and systemically. Since TF is key player in hemostasis, we investigated their protein expression during the late phase of envenomation and how rutin would affect TF. In fact, BjV has been reported to alter TF protein expression in the skin during the acute phase of envenomation (up to 6h) [6, 50], but herein we showed that BjV no longer induced TF alterations at 24 h. This seems a conservative approach by the organism to maintain hemostasis, since TF must be rigidly controlled to avoid systemic activation of coagulation. On the other hand, surprisingly, rutin not only controlled TF activity, particularly in lungs and heart, but also induced marked alterations in protein expression of endogenous and interest proteins.

By Western blotting, rutin itself modified PDI protein expression in liver, lungs, and heart when administered to naive animals (without BjV administration), and thus, we could not evaluate PDI importance during envenomation. However, rutin has various advantageous actions other than in PDI, such as in vascular tonus, endothelium metabolism, and inflammatory reaction, and altogether, independently of the mechanism of action, the current findings support the view that rutin protects animals from the consumptive coagulopathy. Thus, rutin seems a beneficial therapy to be used after *B*. *jararaca* envenomation, although extrapolation of our findings to human patients deserve additional studies and the design of clinical trials.

It is well established that *B*. *jararaca* envenomation induces an acute inflammatory reaction, leading to neutrophilia. Although mice have a lower neutrophil:lymphocyte ratio in blood circulation compared to humans, a mild rise in absolute neutrophil counts in mice, similar to that of human patients, was noticed at 3 h (equivalent to the mean admission time in human patients at the hospital) and at 6 h. [4, 5, 51]. However, rutin *per se* also induced neutrophilia, which may be explained by its ability to reduce rolling, adhesion and transmigration of WBC [52, 53], justifying the increase in the circulating pool of neutrophils in either the presence or absence of BjV. Therefore, rutin could be considered an anti-inflammatory drug, as it prevents neutrophils of committing themselves into the inflammatory reaction, and could be favorably used to diminish the systemic and local lesions resulting from envenomation. However, even though rutin showed the capacity to augment the circulating pool of neutrophils, they do not seem to be primarily implied in the inflammatory reaction induced by BjV, inasmuch as neutrophil depletion does not remarkably interfere in the natural course of the local inflammatory reaction and local hemorrhage induced by BjV in mice [54]. Furthermore, in patients bitten by *B*. *jararaca* snakes, total and differential leukocyte counts do not differ drastically between mild, moderate and severe cases, whose classification depends on the extent and rate of spreading of local swelling at the site of bite, and the occurrence of bleeding and shock [5].

Local injury and bleeding are not exclusively caused by the proteolytic activity of snake venom enzymes, but they also emerge from inflammatory events [32, 55]. Blood platelets continuously survey vascular integrity [56–59], and the coexistence of thrombocytopenia and increased neutrophil migration into tissues during *B*. *jararaca* envenomation [51, 60, 61] might further contribute to blood leakage induced by hemorrhagic toxins and inflammatory mediators at the site of BjV administration [55]. In fact, platelet depletion increases local bleeding, but not edema formation, induced by BjV [62]. Once local bleeding and petechiae development occurs early (< 3h), platelet dysfunction [20] and neutrophil transmigration are likely to have an important role to bleeding development after *B*. *jararaca* envenomation, but further studies are necessary to confirm this hypothesis.

Furthermore, we also observed a mild fall in erythron values induced by *B*. *jararaca* envenomation at 24 h, which was also abrogated by rutin. In humans [5], this fall has been associated with local and systemic bleedings, which in turn is correlated with the hemostatic disturbance. In rats [23], such a fall in RBC was related to aforementioned mechanism and microangiopathic anemia [23]. Nonetheless, our results do not support the view that mice also manifested intravascular hemolysis, however, the results confirm the occurrence of bleeding and hemorrhage in the envenomation, which in turn were mitigated by the use of rutin.

Several studies have reported the use of plant-based or natural antioxidants to inhibit snake venoms *in vitro* or to treat snake envenomation [63, 64]. In fact, the potential of rutin as an ancillary therapy and an anti-hemorrhagic compound was already described in *Bothrops* envenomation studies [63, 65], particularly in a pioneering study from Seba in 1949 [66], which showed that rutin ingestion retarded or abrogated the development of local hemorrhage and inflammatory reactions induced by *Bothrops atrox* venom. Importantly, the administration of rutin to envenomed mice led to a tendency to decrease ROS/RNS levels and increase the antioxidant capacity, which is in accordance with its potent activities of scavenging and neutralizing reactive species, and chelating metal ions [52, 67, 68]. Other beneficial effects reported for rutin, which could alleviate the inflammatory manifestations provoked by *B*. *jararaca* bites include its analgesic and anti-inflammatory effects [17, 69, 70].

A limitation of the current manuscript has been not to investigate the activity of rutin when administered after envenomation or concomitantly with *Bothrops* antivenom. Future studies will certainly focus on its therapeutic use after the venom has initiated the cascade of pathophysiological events, and investigate by which mechanistic action rutin preponderantly inhibits coagulopathy. Using antivenom associated with rutin will be a proof of concept to demonstrate that rutin is really effective in *B*. *jararaca* envenomation.

In conclusion, important impairments of hemostasis, blood cells and redox status were observed during *B*. *jararaca* envenomation. Rutin successfully prevented the development of consumptive coagulopathy, bleeding and local hemorrhage, but failed to mitigate thrombocytopenia. Furthermore, rutin decreased the levels of reactive species and blocked the fall in RBC counts. In lungs and heart, rutin altered TF protein expression and activity. Our results demonstrated that rutin ameliorated coagulation disorders, as well as secondary complications not treated by the antivenom, thus indicating that rutin has indeed a great potential as an ancillary treatment for snakebites. In fact, clinical trials have already been using quercetin and its derivatives as an ancillary therapy to cancer [71–74] and other illness [75], and high oral doses of quercetin (up to 1 g/ day during 12 weeks) showed no toxicity [76]. However, even if quercetin showed no toxicity, further studies are necessary to understand the mechanisms of action and putative side effects of rutin, particularly in humans, prior to evaluating its therapeutic use in snake envenomation.

## Supporting information

S1 Fig Protein expression of β-actin (**a**) and GAPDH (**b**) in liver, lungs, heart and skin in mice at 24 h after treatments. One-way ANOVA was used, followed by Bonferroni *post-hoc* test; # p< 0.05 and # # p< 0.001 when compared to saline control group; * p< 0.05 and ** p< 0.001 when compared to BjV+saline group. Data were expressed as mean ± S.E.M. (n = 5-6/group).
(EPS)

S2 Fig Protein expression of PDI in liver, lungs, heart and skin in mice at 24 h after treatments. One-way ANOVA was used, followed by Bonferroni *post-hoc* test; # p< 0.05 and # # p< 0.001 when compared to saline control group; * p< 0.05 when compared to BjV+saline group. Data were expressed as mean ± S.E.M. (n = 5-6/group).
(EPS)
